# Specifics of depression in epilepsy

**DOI:** 10.1192/j.eurpsy.2022.396

**Published:** 2022-09-01

**Authors:** V. Mitikhin, M. Kuzminova

**Affiliations:** FSBSI “Mental Health Research Centre”, Department Of Mental Health Services, Moscow, Russian Federation

**Keywords:** comorbidity, Depression, epilepsy

## Abstract

**Introduction:**

The strong comorbidity between depression and epilepsy is widely acknowledged. However, depression in epilepsy can manifest atypically, leading to its low detection rate and lack of access to treatment in patients with epilepsy

**Objectives:**

To study the specifics and pattern of depression in epilepsy for its timely diagnosis and therapy and to prevent suicide risk and improve the quality of life in patients with epilepsy

**Methods:**

Clinical, statistical, psychometric. A total of 149 patients, mean age 45.0 ± 11.7 years, 74 males, 75 females, were examined

**Results:**

It was found that depression was manifested in 46.3% of patients before the onset of epileptic seizures, and in 20.8% of patients it developed after treatment with some AEDs. The incidence of symptoms characteristic of depression in epilepsy, such as unstable mood, irritability, euphoria, episodes of pain and sleep disturbances, and its’ impact on the quality of life in patients with epilepsy were analysed. Gender differences were identified for a range of symptoms

**Conclusions:**

The authors expanded their understanding of the clinical specifics of depressive manifestations in patients with epilepsy to allow timely detection and medical and rehabilitative care for these patients

**Disclosure:**

No significant relationships.

